# Confirmation of the absence of local transmission and geographic assignment of imported *falciparum* malaria cases to China using microsatellite panel

**DOI:** 10.1186/s12936-020-03316-3

**Published:** 2020-07-13

**Authors:** Yaobao Liu, Sofonias K. Tessema, Maxwell Murphy, Sui Xu, Alanna Schwartz, Weiming Wang, Yuanyuan Cao, Feng Lu, Jianxia Tang, Yaping Gu, Guoding Zhu, Huayun Zhou, Qi Gao, Rui Huang, Jun Cao, Bryan Greenhouse

**Affiliations:** 1 National Health Commission Key Laboratory of Parasitic Disease Control and Prevention, Jiangsu Provincial Key Laboratory on Parasite and Vector Control Technology, Jiangsu Institute of Parasitic Diseases, Wuxi, Jiangsu China; 2 Medical College of Soochow University, Suzhou, Jiangsu China; 3 EPPI Center Program, Division of HIV, Infectious Diseases, and Global Medicine, Department of Medicine, University of California San Francisco, San Francisco, CA USA; 4 Center for Global Health, School of Public Health, Nanjing Medical University, Nanjing, China; 5 Public Health Research Center, Jiangnan University, Wuxi, China; 6 Chan Zuckerberg Biohub, San Francisco, CA USA; 7 Department of Parasitology, Institute of Translational Medicine, Medical College, Yangzhou University, Jiangsu Key Laboratory of Experimental & Translational Non- coding RNA Research, Yangzhou, Jiangsu China

**Keywords:** Malaria, Imported malaria, Local transmission, Jiangsu, China, Microsatellite genotyping, Geographic assignment

## Abstract

**Background:**

Current methods to classify local and imported malaria infections depend primarily on patient travel history, which can have limited accuracy. Genotyping has been investigated as a complementary approach to track the spread of malaria and identify the origin of imported infections.

**Methods:**

An extended panel of 26 microsatellites (16 new microsatellites) for *Plasmodium falciparum* was evaluated in 602 imported infections from 26 sub-Saharan African countries to the Jiangsu Province of People’s Republic of China. The potential of the 26 microsatellite markers to assign imported parasites to their geographic origin was assessed using a Bayesian method with Markov Chain Monte Carlo (MCMC) as implemented in the program Smoothed and Continuous Assignments (SCAT) with a modification to incorporate haploid genotype data.

**Results:**

The newly designed microsatellites were polymorphic and are not in linkage disequilibrium with the existing microsatellites, supporting previous findings of high rate of recombination in sub-Saharan Africa. Consistent with epidemiology inferred from patients’ travel history, no evidence for local transmission was found; nearly all genetically related infections were identified in people who travelled to the same country near the same time. The smoothing assignment method assigned imported cases to their likely geographic origin with an accuracy (Angola: 59%; Nigeria: 51%; Equatorial Guinea: 40%) higher than would be achieved at random, reaching statistical significance for Angola and Equatorial Guinea.

**Conclusions:**

Genotyping using an extended microsatellite panel is valuable for malaria case classification and programme evaluation in an elimination setting. A Bayesian method for assigning geographic origin of mammals based on genetic data was adapted for malaria and showed potential for identification of the origin of imported infections.

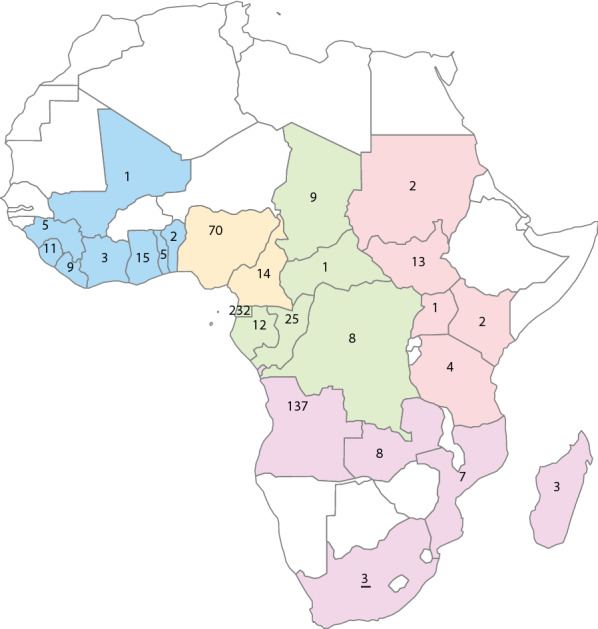

## Background

China has made enormous progress in controlling malaria and has an ambitious goal of malaria elimination by 2020 [1]. There has been a decline in indigenous malaria cases, and most areas of the country are now in the prevention of re-introduction phase [1]. Meanwhile, with increasing global human movement, imported malaria has become a major challenge for elimination in many regions [2, 3]. In countries approaching elimination, imported cases account for the majority of reported cases and threaten resurgence of transmission in receptive areas due to the persistence of malaria vectors [4, 5].

Imported malaria has significantly increased in China over the past few years, causing 19,574 cases and 98 deaths (11.3 per 1000 cases) over the 5-year period 2011–2015 [6]. The proportion of cases which were imported reached 98.2% (3022/3078) and 98.8% (3253/3294) of all cases in 2014 and 2015, respectively [7]. Most imported cases are caused by *Plasmodium falciparum* and originate in sub-Saharan Africa from labour-related Chinese travellers [8, 9]. Recent studies showed a significant association between the number of imported cases and the amount of China’s financial investment and malaria transmission intensity in sub-Saharan African countries [6]. These investments are predicted to expand in scope and volume and increase the risk of malaria importation [10].

Local transmission of *P. falciparum* has been interrupted in the Jiangsu Province of China since 1989 [11]. However, this success is vulnerable to a possible resurgence of local transmission given the potential receptivity and increasing volume of importation. This vulnerability has been demonstrated by studies that showed agreement between the location of imported cases and the environmental suitability for malaria transmission [12]. Genetic characterization of imported cases could potentially give insight on the origin of parasites, when this is not clear based on travel history, as well as detect any evidence of local transmission.

Despite recent advances in genome sequencing of malaria parasites [13], microsatellite markers remain an important source of data to understand the genetic diversity and relatedness of parasite populations and understanding of transmission dynamics [14, 15]. Microsatellite markers are abundant in the *P. falciparum* genome [16], and tend to be more reliably variable in multiple populations than other markers. However only 10 microsatellite loci are frequently used in most studies on the diversity and genetic relatedness of parasite populations [17, 18]. This study designed 16 additional microsatellite markers flanking the most commonly used 10 microsatellite markers [17]. The expanded panel was used to characterize 602 *P. falciparum* isolates collected from Chinese returnees from 26 sub-Saharan African countries to Jiangsu Province of China. The genetic, spatial and temporal relatedness of imported infections were assessed, and population assignment methods were used to identify the geographic origin of the imported cases.

## Methods

### Sample and data collection

Venous blood samples were collected from symptomatic patients presented between September 2011 and March 2015 to a local hospital or centre for disease control (CDC) in 13 prefectures of Jiangsu Province of China. The detailed epidemiological data of malaria cases, such as age, gender, travel history, time spent in African countries, and the number of symptomatic episodes were collected from the web-based China Information System for Disease Control and Prevention (CISDCP) and individual case epidemiological investigation reports. According to the Technical Scheme of China Malaria Elimination [19], imported malaria cases were identified based on the travel history of the patient (travel within the previous month to a malaria-endemic country) and the last country visited with ongoing malaria transmission was taken as the potential location of infection. Otherwise, the case was classified as locally acquired case. All participants gave written informed consent and microscopy-positive samples were submitted to the Jiangsu Provincial Reference Laboratory of Malaria Diagnosis for confirmation by microscopy and species-specific nested polymerase chain reaction (PCR). Species-specific PCR was done as described previously [20, 21].

### DNA extraction and microsatellite genotyping

Genomic DNA was extracted from 200 μL of blood with the QIAamp blood DNA extraction kit (Qiagen, Crawley, UK) according to manufacturer’s instructions. Ten extensively studied microsatellite markers (Polyα, TA81, TA87, TA1, TA109, TA40, ARA2, PfG377, PfPk2, TA60), referred as the original panel, were used to guide and select additional tandem repeats in the up- and down-stream flanking regions [17, 18]. After excluding microsatellite markers that showed inconsistent amplification and low heterozygosity, 16 additional microsatellite markers were designed and included in this analysis (see Additional file 1). The 16 newly identified markers flank the 10 microsatellite markers, allowing evaluation of short-range haplotypes, here termed ‘meta-loci’.

A two-round PCR protocol was used to amplify the 26 microsatellite loci, referred to as the extended microsatellite panel. The multiplex primary PCR was performed in 4 groups using 2 different PCR conditions. One μL of the amplified product was then used as a template for each individual secondary PCR **(**Primer sequences, PCR conditions are provided in Additional file 2). The labelled PCR products were then diluted and sized by denaturing capillary electrophoresis on an ABI 3730XL analyzer with GeneScan™ 400HD ROX™ Size Standard (Thermo Fisher Scientific). The resulting electropherograms were analysed using microSPAT [22], which identifies true peaks from artifacts using a probability-based filter. All results were verified manually. Only peaks with ≥ 300 relative fluorescence units were filtered and processed for allele calling.

### Multiplicity of infection

Infections containing more than one allele for at least two markers were classified as multi-clone infections. The multiplicity of infection (MOI) for a given sample was defined as the maximum number of alleles observed at any of the 26 loci investigated, after removing the locus with the highest number to decrease susceptibility to outliers due to PCR artifact. Analyses were carried out using the original panel, the extended panel, and the extended panel grouped as meta-loci. For geographic analysis, samples were grouped into five regions including East Africa (Kenya, South Sudan, Sudan, Tanzania, Uganda), Central Africa (Central African Republic, Chad, Democratic Republic of the Congo, Equatorial Guinea, Gabon, Republic of the Congo), Central-West Africa (Cameroon, Nigeria), West Africa (Benin, Côte d’Ivoire, Ghana, Guinea, Liberia, Mali, Sierra Leone, Togo) and Southern Africa (Angola, Madagascar, Mozambique, South Africa, Zambia).

### Population genetic indices

Population-level genetic diversity was characterized by expected heterozygosity (*H*_E_) and allelic richness (*R*_S_) normalized by the smallest sample size. These two parameters were used to assess the level of genetic polymorphism at each locus and determine the overall genetic diversity. *H*_E_ was calculated using the formula, *H*_E_ = [n/(n − 1)][1 − ∑p^2^] where *n* is the number of genotyped samples and *p* is the frequency of each allele at a locus. The pair-wise *Jost’s D* [23] and *G*_ST_ [24] metrics were used to estimate the genetic differentiation between pairs of populations. Multi-locus linkage disequilibrium (LD) was measured by the standardized index of association (*I*_A_^S^) using the web-based LIAN 3.5 software [25]. Under the null hypothesis of linkage equilibrium, the significance of the *I*_A_^S^ estimates was assessed using 10,000 random permutations of the data.

### Population structure and genetic relatedness

Spatial and temporal population structure of the imported cases were inferred using the admixture model as implemented in MavericK v1.0 [26]. Genetic distance between isolates is often determined based on alleles detected in monoclonal infections or taking into account the dominant allele identified for each locus. An approach to determine a modified identity by state (IBS) metric [27] between each pair of isolates was employed. IBS was calculated based on the number of alleles shared between isolates, rather than of their haplotypes, allowing for the inclusion of mono- and polyclonal samples. Pair-wise IBS was determined as described elsewhere [28, 29]. Using this distance metric ($1 - IBS$), a neighbour-joining tree and multidimensional scaling were calculated.

### Population assignment

The potential of the 26 microsatellite markers to assign imported parasites to their geographic origin was assessed using a Bayesian method with MCMC as implemented in the program Smoothed and Continuous Assignments (SCAT) [30] with a modification to incorporate haploid genotype data. Following validation of the assignment tool, the assignment accuracy of imported cases to their likely origins in sub-Saharan Africa was investigated. A leave-one-out cross-validation procedure was conducted in 243 monoclonal *P. falciparum* isolates imported from Angola (n = 78), Equatorial Guinea (n = 124) and Nigeria (n = 41), the only three countries with at least 40 single-clone infections in this study. In these analyses, each sample was treated as the sample whose location was unknown, whereas the other samples were assumed to have a known location (i.e., the centroid location of the respective countries). Smoothing and continuous assignment methods were used to assign imported cases to their geographic origin as described previously [30]. Three independent assignment runs were performed, each starting with a different seed. For each run the first 2000 iterations were discarded as burn-in, and every 10th of the following 3000 iterations were stored, resulting in 300 putative locations of origin for each isolate. The range of allowable locations for the continuous assignment of individuals was specified by creating a boundary around sub-Saharan Africa. The assignment tests were also conducted for randomly generated alleles to create a null distribution. For the continuous assignment method, the median distance of the assigned isolates was calculated from the centroid location of the country. The assignment accuracy of the smoothing method was evaluated by calculating the average accuracy for each run (i.e., the proportions of correctly assigned individuals within each country).

## Results

### Epidemiologic profile of imported malaria in Jiangsu Province, China

**Fig. 1:**
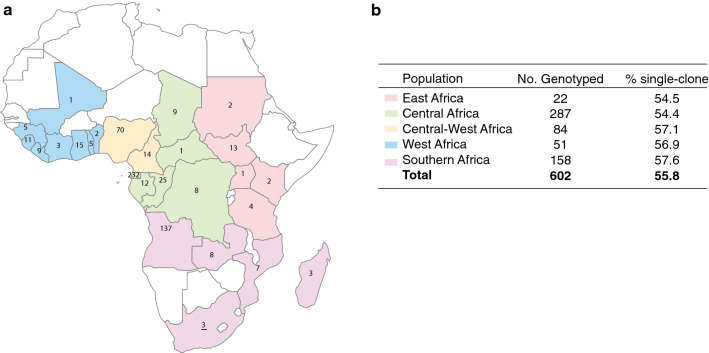
Origins of imported malaria cases from five regions of sub-Saharan Africa to the Jiangsu Province of China. **a** Number of samples from each country on the map. **b** The total number of genotyped samples per region and percentage of single-clone infections

A total of 946 laboratory-confirmed falciparum malaria cases was identified in Chinese travellers returned from 26 African countries to Jiangsu Province over a five-year period: 2011 (57), 2012 (109), 2013 (332), 2014 (342), and 2015 (106). Of the 946 imported cases, 627 falciparum malaria cases were available for genotyping using the extended microsatellite panel; 96% (602) were successfully genotyped at 22 or more of the 26 loci. The majority of the genotyped cases were imported from Equatorial Guinea (232), Angola (137) and Nigeria (70). The median age of patients was 44 years (range: 15–61 years), and males accounted for 98.8% of the imported cases. The imported cases were grouped into five regions based on the geographic proximity of the 26 African countries (East, Central, Central-West, West and Southern Africa) (Fig. 1).

The travellers who acquired *P. falciparum* spent an average of 351 days (IQR: 196-655 days) in the respective African countries; 46.8% of the travellers reported at least one symptomatic episode during their stay in Africa with an average of 2.5 episodes. Chinese travellers to southern Africa had the lowest number of reported episodes (mean = 1.2), while travellers to central Africa had the highest number of reported episodes (mean = 3.9). The median parasite density for all imported cases was 19,820 parasites per µL (Table 1). As might be expected due to development of partial immunity, individuals with reported malaria episodes during their stay in Africa had significantly lower parasite density upon presentation in China (median = 12,920 (IQR: 3476–48,966) parasites per µL) than those who did not (median = 37,600 (IQR: 6500–118,765) parasites per µL; *p *= 0.0003, Wilcoxon rank sum test) and spent more time in sub-Saharan African countries (401 vs 280 days, p < 0.001).

**Table 1:** Summary of imported malaria cases from sub-Saharan Africa to Jiangsu Province, China

Regions	Year reported	No. samples		Duration of stay (median [IQR])	No. reported malaria episodes mean [sd]	Parasite density (× 1,000) median [IQR])
		Total	Genotyped			
East Africa	2013–2014	25	22	286 [182, 390]	1.9 [1.8]	8.6 [3.8, 34.6]
Central Africa	2011–2015	512	287	394 [194, 688]	3.9 [4.6]	13.9 [3.6, 80]
Central-West Africa	2013–2014	113	84	254 [138, 371]	1.9 [2.2]	21 [4.5, 77]
West Africa	2013–2014	63	51	232 [96, 348]	1.8 [2.6]	27 [5.7, 75.7]
Southern Africa	2013–2014	233	158	471 [268, 748]	1.2 [1.3]	30 [6.6, 100]
Total	2011–2015	946	602	351 [196, 655]	2.5 [3.5]	19.8 [4.6, 81.1]

### Description and characteristics of the extended microsatellite panel

**Fig. 2:**
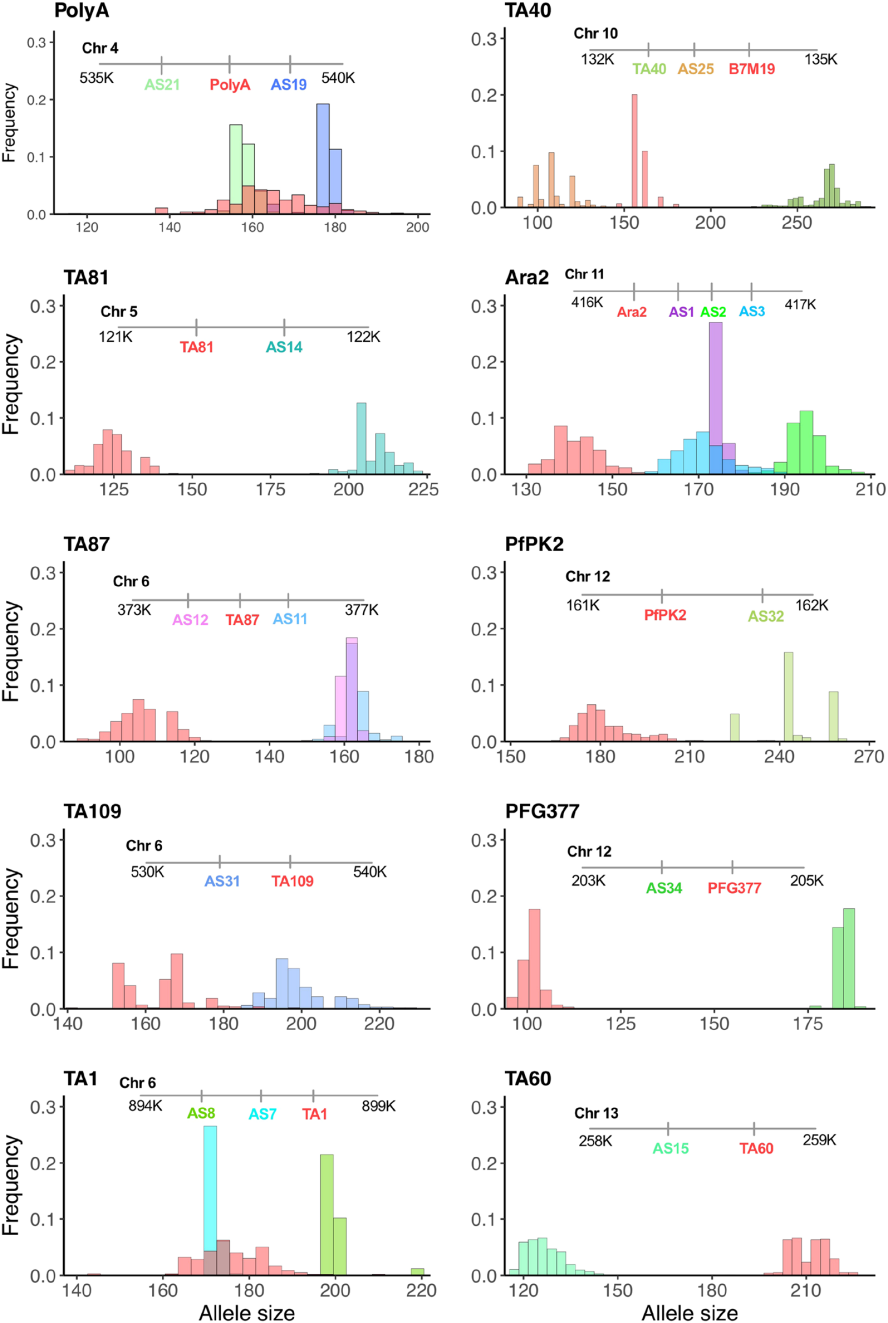
Allele size and frequency distribution of alleles for the extended microsatellites panel. The colour of locus name corresponds to the colour of the column

The extended microsatellite panel includes 16 newly identified loci flanking 10 previously described microsatellite panels [17]. The 26 microsatellites were evaluated for their utility as genotyping markers. For single clone infections, short-range multilocus haplotypes were constructed from flanking microsatellite markers to create a meta-locus for each of the 10 genomic regions (see Additional file 1). The chromosomal location and frequency distribution of alleles are shown in Fig. 2 (the colour of locus name corresponds to the colour of the column). All the newly identified microsatellite markers were neutral and polymorphic in this study population. The number of unique alleles per locus ranged from 6 (AS1 and AS12) to 21 (AS25) with a mean expected heterozygosity of 0.65 (range: 0.38–0.85) and with 13/16 new markers exhibiting an expected heterozygosity greater than 0.5 (see Additional files 3, 4).

Multilocus LD was calculated using the standardized index of association (*I*_*A*_^*S*^), excluding samples with polyclonal infections. Overall, low LD was detected in pair-wise comparisons between the 26 loci in the extended panel (*I*_*A*_^*S*^ = 0.006, range: 0–0.05). Associations between loci within the same chromosome or meta-locus were not significantly different from those with markers located on a different chromosome or a meta-locus, consistent with the high, expected rate of recombination in parasite populations of sub-Saharan Africa (see Additional file 5). A very low yet significant multi-locus LD was observed in all five regional populations of sub-Saharan Africa for the 26 loci (*I*_*A*_^*S*^ = 0.004, *p* < 0.001); 10 loci (*I*_*A*_^*S*^ = 0.003, *p* = 0.015) and meta-loci (*I*_*A*_^*S*^ = 0.004, *p* < 0.001) (see Additional file 5). Due to the low pair-wise LD between the extended microsatellite loci, including between flanking microsatellites, the rest of the analyses were performed by considering each locus as an independent unit.

### Moderate complexity and high genetic diversity of imported cases

The successfully genotyped isolates had a mean MOI of 1.6 (range: 1–5), and 59% were single-clone infections (see Additional file 6). Mean MOI and proportions of multi-clone infections were not significantly different among infections imported from the five regions of Africa. There was no association between MOI and parasite density (r = − 0.007, p = 0.87). Furthermore, there was no difference in the mean MOI of those who reported malaria during their stay in Africa (mean MOI = 1.64) and those who did not (mean MOI = 1.60, p = 0.71).

Allelic richness (*R*_S_*)* and expected heterozygosity (*H*_E_) of the monoclonal samples were compared among the five populations using the extended and original microsatellite panels [17, 18]. Across all samples, a total of 375 and 194 unique alleles were identified from the 26 and 10 loci, respectively. There was no significant difference in allelic richness and expected heterozygosity between infections imported from the five regions of sub-Saharan Africa (Table 2).

**Table 2:** Genetic diversity and allelic richness of 336 imported monoclonal *Plasmodium falciparum* isolates from five regional sub-populations of Africa

Population	No. samples	Extended panel (n = 26 loci)		Original panel (n = 10 loci)	
		R_S_ ± SE^a^	H_E_ ± SE^b^	R_S_ ± SE	H_E_ ± SE
East Africa	12	4.2 ± 0.30	0.68 ± 0.038	3.12 ± 0.26	0.82 ± 0.033
Central Africa	156	4.1 ± 0.30	0.72 ± 0.035	3.31 ± 0.28	0.84 ± 0.022
Central West Africa	48	3.8 ± 0.28	0.70 ± 0.038	2.77 ± 0.22	0.83 ± 0.028
West Africa	29	4.0 ± 0.28	0.71 ± 0.034	3.03 ± 0.25	0.83 ± 0.025
Southern Africa	91	3.9 ± 0.29	0.72 ± 0.033	2.91 ± 0.27	0.85 ± 0.022
Total	336	4.0 ± 0.13	0.71 ± 0.016	3.02 ± 0.11	0.83 ± 0.011

^a^*R*_S_–Allelic richness based on the minimum sample size of 12 individuals (East Africa) and ^b^*H*_E_ is the mean expected heterozygosity

### Low to moderate geographical differentiation of imported infections

Low to moderate genetic differentiations were observed between the five parasite sub-populations for the extended (G_ST_ = 0.004–0.017*, Jost’s* D =0.019–0.075) and original microsatellite panels (G_ST_ = 0.003–0.017*, Jost’s* D =0.037–0.155) (Table 3). The least differentiation was observed between parasite populations from Central and Central-West Africa (G_ST_ = 0.004), Central and West Africa (G_ST_ = 0.008) and Central and Southern Africa (G_ST_ = 0.006). *Jost’s D* values among the five regions indicated a wide range of private alleles ranging from 1.9 to 7.5% in the 26 loci and 3.7 to 15.5% in the 10 loci.

**Table 3:** Estimates of genetic differentiation among parasite isolates imported from five regional sub-populations of sub-Saharan Africa (*upper triangle is Jost’s D and bottom is G*_*ST*_*)*

Regions	East Africa	Central Africa	Central-West Africa	West Africa	Southern Africa
A. Extended panel (n = 26 loci)					
East Africa	–	0.046	0.05	0.054*	0.075*
Central Africa	0.011	–	0.019	0.038	0.031
Central-West Africa	0.012	0.004	–	0.055	0.046
West Africa	0.017*	0.008	0.013	–	0.058
Southern Africa	0.017*	0.006	0.01	0.013	–
B. Original panel (n = 10 loci)					
East Africa	–	0.105	0.104	0.129*	0.155*
Central Africa	0.012	–	0.038	0.037	0.035
Central-West Africa	0.012	0.004	–	0.08	0.051
West Africa	0.016*	0.004	0.009	–	0.071
Southern Africa	0.017*	0.003	0.005	0.007	–

*Significant genetic differentiations (*p *< *0.05*)

### Imported falciparum malaria cases were diverse and not structured

**Fig. 3:**
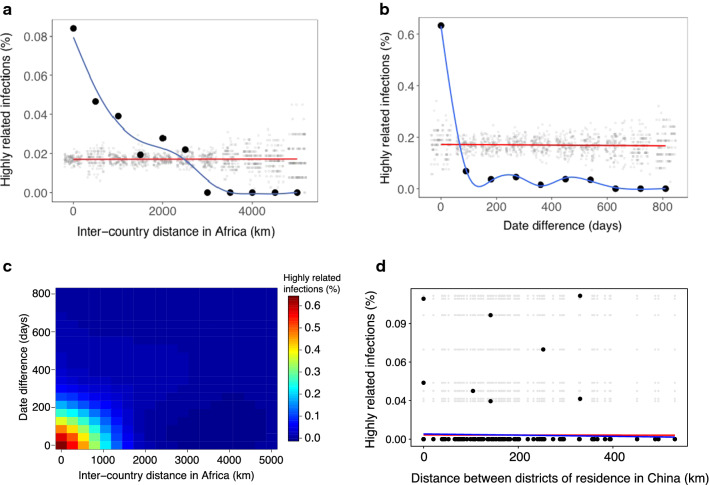
Characterization of highly related infections. **a** Relationship between the proportion of highly related infections and distance between destination countries in Africa. **b** Relationship between the proportion of highly related infections and date difference in the reporting of malaria cases in Jiangsu, China**. c** Relationship between the proportion of highly related infections, distance and reporting time. Genetically related infections were usually imported from shared trips to a similar destination. **d** Relationship between the proportion of highly related infections and distance between residence district in Jiangsu, China. For panels A, B and D, black dots and blue lines indicate the observed and fitted data, respectively. The grey dots and red lines indicate a null distribution from a permutation test

Pair-wise comparison of genetic relatedness between all pairs of infections revealed that most infections were genetically unrelated (median genetic relatedness = 0.29 (IQR: 0.24–0.34)) (see Additional file 7). Similarly, admixture model-based analyses did not find spatial and temporal structure in the imported infections (see Additional file 7). However, against this background, 39 highly related pairs of imported infections (genetic relatedness ≥ 0.6) were detected. All related pairs had travel history consistent with importation from the same or a nearby country (Fig. 3a). On average, highly related infections were imported and reported in China within 59 days (IQR: 22–202 days), compared to 214 days (IQR: 98–371 days) for unrelated infections (Fig. 3b, c). Similarly, highly related infections tended to be imported from a similar region (645 km (IQR: 0–1793 km)) than unrelated infections (1827 km (IQR: 645–2437 km) (p = 0.0003) (Fig. 3c). No relationship between parasite relatedness and patient residence in Jiangsu, China was observed (Fig. 3d), supporting the absence of local transmission.

### Geographic assignment of imported infections

**Fig. 4:**
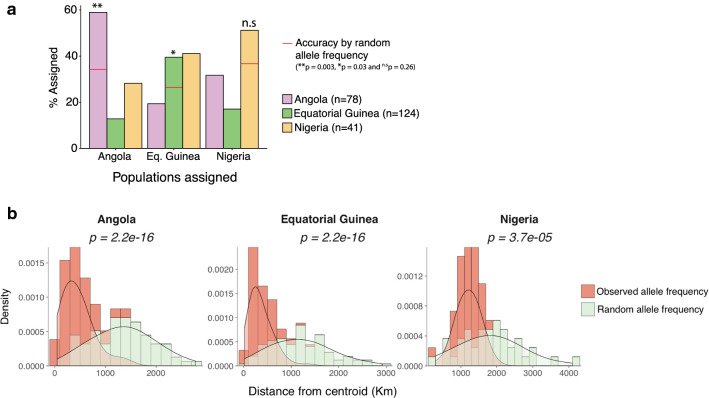
Population assignment of 243 imported *Plasmodium falciparum* cases from Angola, Equatorial Guinea and Nigeria. **a** Assignment accuracy for imported cases using the smoothing-based assignment method. **b** Estimated locations of origin of 243 imported *P. falciparum* cases using the continuous-based assignment method

While unsupervised clustering methods were unable to detect any significant structure in the genetic data, methods were also evaluated for assignment of geographic origin using training data. The smoothing assignment method assigned imported cases to their likely geographic origin with an accuracy of 59, 51 and 40% for Angola, Nigeria and Equatorial Guinea, respectively. Despite the relatively low accuracy, the assignment accuracy was higher than would be achieved at random, reaching statistical significance for Angola and Equatorial Guinea (Fig. 4a). Using the continuous assignment method, the Euclidian distances between the estimated origin of each sample from the centroid location of the respective countries was determined with observed and randomly generated allele frequencies. Overall, 50% of the samples were assigned within 390 km of the centroid location of the origin country. The median distance was 391 km for Angola, 297 km for Equatorial Guinea, and 1197 km for Nigeria, all significantly closer than the expected assignment distance from randomly generated genotypes (Fig. 4b). While the genetic data available in this study did not allow precise assignment, these findings highlight the potential of genetic assignment methods to provide more information on the geographic origin of imported infections than unsupervised clustering methods.

## Discussion

Genetic surveillance has the potential to provide useful information for malaria transmission epidemiology, particularly in the elimination context where detecting ongoing local transmission and identifying the origin of imported infections may be relevant. Using an extended panel of microsatellite markers, the results reveal importation of diverse parasite populations from different regions of sub-Saharan Africa to Jiangsu Province of China. Reassuringly, and consistent with epidemiology inferred from the travel history of cases, no evidence was found for local transmission: nearly all closely related infections were identified in people who travelled to the same or neighbouring country near the same time. While the absence of local transmission has been well documented in Jiangsu, these results highlight the potential utility of related methods for surveillance in areas where the lack of transmission is less clear, e.g., in areas that have only recently achieved elimination and/or where surveillance systems are not as robust. The absence of malaria transmission in Jiangsu Province is probably due to the limited receptivity of falciparum malaria [11, 31] as well as the implementation of effective surveillance and response strategies such as ‘1-3-7’ strategy [32]; however, persistent importation could result in a re-emergence of local transmission [33], highlighting the need to sustain stringent surveillance efforts in receptive areas of China [32, 34].

Chinese travellers importing malaria spent an average of 1 year in respective African countries, and of those, 47% reported symptomatic malaria episodes during their stay. Despite the duration of stay and the risk of infections, genetic characterization of imported cases revealed a limited number of genotypes per imported infection despite the high diversity of genotypes in the local parasite populations, suggesting limited superinfection of the travellers. In contrast to reports characterizing the diversity of locally detected cases, the average number of distinct genotypes per infection did not differ among cases imported from different regions of sub-Saharan Africa [17, 35, 36].

Due to the physical proximity of the newly designed markers with the existing loci, strong pair-wise LD between flanking microsatellites was expected, however the pair-wise LD was very low even among loci within 1000 bp on the same chromosome, suggesting very high rate of recombination in *P. falciparum* parasites in sub-Saharan Africa as described previously [37, 38]. This observation is further evidenced by high genetic diversity in imported infection, similar to the level reported from local parasite populations [35, 39, 40] and the lack of detectable geographic clusters. Furthermore, imported infections exhibited significant differentiation only in those imported from East *versus* West and East *versus* Southern Africa countries, consistent with previous reports [17, 38].

Multi-locus genotyping data, combined with statistical methods of inference, have been employed to address key questions of genetic identity and population membership, even when the overall level of genetic differentiation among populations is low [30, 41–43]. Such methods have been commonly used to assess temporal and spatial clustering of *P. falciparum* as well as *Plasmodium vivax* populations at continental as well as village level [15, 44–47]. However, these methods have not been standardized for the classification of imported *versus* local malaria cases. In this study, it was relatively trivial to document the lack of closely related infections, given the use of highly diverse genetic markers which provided robust data even in polyclonal infections, the setting of a robust surveillance system capturing all or nearly all cases, and importation of parasites from diverse, relatively high transmission areas. However, while appropriate genetic markers are available to choose, the other conditions are not always met, highlighting the need to develop more formal methods for distinguishing local *versus* imported cases.

Current methods rely on reported travel history to identify the origin of imported infections, but such data are often incomplete in areas where surveillance is weak. The strong surveillance system in China allowed evaluation of the value of genetic data to accurately assign imported cases to their geographic origins. The origins of infections were able to be identified better than expected by chance, highlighting the potential of genetic data to identify and assign the geographic origins of imported infections for malaria case classification and programme evaluation in an elimination setting. However, the assignment in this case was not accurate enough to be useful for surveillance. The lower assignment accuracy observed in this study could be improved by (1) specifically identifying a panel of geographically informative loci; (2) developing sensitive and high throughput laboratory methods to genotype these loci; and, (3) development of formalized analytical tools that can incorporate polyclonal infections to perform accurate classification of local, imported and introduced cases as well as identify the origin of imported infections. In addition, the further practical application of the approach needs to consider the ability of laboratory testing and data analysis in the settings.

Expanding the genetic epidemiology toolkit is a timely task to obtain a better insight into the spatial and temporal dynamics of transmission as well as for accurate classification of local and imported infections. Using an expanded microsatellite panel, the absence of local transmission in Jiangsu Province was confirmed and the potential for genetic data to identify the geographic origin of imported malaria infections was demonstrated. More formalized methods would allow surveillance systems to track infections and develop targeted policies to limit the risk of re-introduction of falciparum malaria in eliminating countries. The limitation of this study is that samples were collected from imported cases and their geographic origins in Africa were concentrated in three regions/countries, therefore the assign approach has not been verified in a larger geographic scope and the value of its routine application requires further validation.

## Conclusions

The newly designed microsatellites were polymorphic and no evidence for local falciparum malaria transmission was found based on the extended microsatellite panel in Jiangsu Province of China. Genotyping is valuable for malaria case classification and programme evaluation in an elimination setting. A Bayesian method for assigning geographic origin of mammals based on genetic data showed potential for identification of the origin of imported malaria infections.

## Supplementary information

**Additional file 1.** Description of the 16 newly designed and 10 existing microsatellite markers used in this study

**Additional file 2.** Primer sequences, PCR master mixes and cycling conditions for the primary and secondary PCRs A. List of primers B. Primary PCR C. Secondary PCR D. Dilution and CE.

**Additional file 3.** Genetic diversity and the number of unique alleles in 336 imported monoclonal *Plasmodium falciparum* isolates from five regional populations of sub-Saharan Africa. New microsatellite markers are highlighted in grey.

**Additional file 4.** Relationship between the number of unique alleles and expected heterozygosity in 602 *Plasmodium falciparum* cases imported to Jiangsu Province from 26 African countries. Previously described markers are shown in red and new markers are shown in blue.

**Additional file 5.** Pair-wise linkage disequilibrium between 26 microsatellite markers. Pair-wise index of associations (IA) is indicated. B. Estimates of multi-locus linkage disequilibrium in 336 *Plasmodium falciparum* isolates imported from five different regions of sub-Saharan Africa to Jiangsu Province, China IAS is the standardized index of association.

**Additional file 6.** Mean multiplicity of infection (MOI) and percentage of polyclonal samples by the geographic origin of imported infections to the Jiangsu Province, China.

**Additional file 7**. A. Neighbour-joining tree showing genetic relatedness of 602 imported *Plasmodium falciparum* isolates, branches are coloured according to region of origin of the imported case. B. Plot of second principal component against the first, computed from a multidimensional scaling based on the same distance matrix used for the tree shown in panel A.

## Data Availability

Not applicable.

## Abbreviations

MCMC: Markov chain monte carlo
SCAT: Smoothed and continuous assignments
CDC: Center for disease control
PCR: Polymerase chain reaction (PCR)
MOI: Multiplicity of infection
*H*_E_: Heterozygosity
*R*_S_: Allelic richness
LD: Linkage disequilibrium
*I*_A_^S^: Standardized index of association
IBS: Identity by state
