# Supplementary material for: Confirmation of the absence of local transmission and geographic assignment of imported falciparum malaria cases to China using microsatellite panel
Source: Malar J. 2020 Jul 13;19:244. doi: 10.1186/s12936-020-03316-3 (PMC7359230; doi:10.1186/s12936-020-03316-3)
Supplement: Supplementary file 1 — Additional file 1. Description of the 16 newly designed and 10 existing microsatellite markers used in this study [file 12936_2020_3316_MOESM1_ESM.docx]

**Table S1.** Description of the 16 newly designed and 10 existing microsatellite markers used in this study

| **Microsatellite Marker** | **Home MS Marker** | **Chromosome** | **Start location#** | **Size Range (bp)** | **Reference** |
| --- | --- | --- | --- | --- | --- |
| AS21 | Polyα | 4 | 535430 | 143-195 | New |
| Polyα |  |  | 539030 | 95-210 | Anderson *et al.,* 2000 |
| AS19 |  |  | 540330 | 125-205 | New |
| TA81 | TA81 | 5 | 1214314 | 103-166 | Anderson *et al.,* 2000 |
| AS14 |  |  | 1218914 | 184-235 | New |
| AS12 | TA87 | 6 | 373327 | 137-186 | New |
| TA87 |  |  | 374727 | 80-142 | Anderson *et al.,* 2000 |
| AS11 |  |  | 377427 | 140-189 | New |
| AS31 | TA109 | 6 | 797386 | 146-234 | New |
| TA109 |  |  | 800986 | 135-235 | Anderson *et al.,* 2000 |
| AS8 | TA1 | 6 | 894144 | 168-226 | New |
| AS7 |  |  | 899270 | 150-199 | New |
| TA1 |  |  | 899844 | 130-230 | Anderson *et al.,* 2000 |
| TA40 | TA40 | 10 | 1322577 | 200-310 | Anderson *et al.,* 2000 |
| AS25 |  |  | 1324577 | 64-152 | New |
| B7M19 |  |  | 1356141 | 133-192 | New |
| ARA2 | ARA2 | 11 | 416295 | 117-166 | Anderson *et al.,* 2000 |
| AS1 |  |  | 416481 | 160-190 | New |
| AS2 |  |  | 416821 | 178-218 | New |
| AS3 |  |  | 417695 | 146-198 | New |
| PfPK2 | PfPK2 | 12 | 1611190 | 140-235 | Anderson *et al.,* 2000 |
| AS32 |  |  | 1623190 | 190-295 | New |
| AS34 | PfG377 | 12 | 2034910 | 149-204 | New |
| PFG377 |  |  | 2045810 | 86-131 | Anderson *et al.,* 2000 |
| AS15 | TA60 | 13 | 2584124 | 107-159 | New |
| TA60 |  |  | 2584963 | 179-255 | Anderson *et al.,* 2000 |

#Sequences of the 3D7 genome were obtained from PlasmoDB version
