# Supplementary material for: Confirmation of the absence of local transmission and geographic assignment of imported falciparum malaria cases to China using microsatellite panel
Source: Malar J. 2020 Jul 13;19:244. doi: 10.1186/s12936-020-03316-3 (PMC7359230; doi:10.1186/s12936-020-03316-3)
Supplement: Supplementary file 3 — Additional file 3. Genetic diversity and the number of unique alleles in 336 imported monoclonal Plasmodium falciparum isolates from five regional populations of sub-Saharan Africa. New microsatellite markers are highlighted in grey. [file 12936_2020_3316_MOESM3_ESM.docx]

**Table S3.** Genetic diversity and the number of unique alleles in 336 imported monoclonal *P. falciparum* isolates from 5 regional populations of sub-Sharan Africa. New microsatellite markers are highlighted in grey.

| **^*^*Chr*** | **Metalocus** | **Locus** | **All**  **(n=336)** | | **East Africa (n=12)** | | **Central Africa (n=156 )** | | | **Central-West Africa (n=48)** | | | **West Africa (n=29)** | | | **Southern Africa (n=91)** | | |
| --- | --- | --- | --- | --- | --- | --- | --- | --- | --- | --- | --- | --- | --- | --- | --- | --- | --- | --- |
|  |  |  |  |  |  |  |  |  |  |  |  |  |  |  |  |  |  |  |
|  |  |  | ^†^***A*** | ^‡^***He*** | ***A*** | ***He*** | ***A*** | ***He*** | ***A*** | | ***He*** | ***A*** | | ***He*** | ***A*** | | ***He*** |  |
| 4 | Polyα | AS21 | 7 | 0.39 | 2 | 0.17 | 7 | 0.34 | 2 | | 0.16 | 4 | | 0.46 | 4 | | 0.53 |  |
|  |  | Polyα | 22 | 0.92 | 8 | 0.95 | 19 | 0.92 | 16 | | 0.92 | 10 | | 0.88 | 18 | | 0.92 |  |
|  |  | AS19 | 5 | 0.55 | 3 | 0.59 | 5 | 0.53 | 4 | | 0.53 | 3 | | 0.54 | 4 | | 0.58 |  |
| 5 | TA81 | TA81 | 13 | 0.85 | 6 | 0.87 | 13 | 0.84 | 10 | | 0.86 | 7 | | 0.81 | 9 | | 0.87 |  |
|  |  | AS14 | 12 | 0.78 | 4 | 0.64 | 12 | 0.82 | 7 | | 0.76 | 5 | | 0.58 | 10 | | 0.76 |  |
| 6 | TA87 | AS12 | 6 | 0.51 | 2 | 0.41 | 4 | 0.5 | 4 | | 0.57 | 4 | | 0.57 | 5 | | 0.45 |  |
|  |  | TA87 | 16 | 0.86 | 5 | 0.85 | 14 | 0.87 | 9 | | 0.83 | 8 | | 0.87 | 11 | | 0.86 |  |
|  |  | AS11 | 9 | 0.65 | 4 | 0.74 | 9 | 0.67 | 6 | | 0.56 | 3 | | 0.65 | 8 | | 0.67 |  |
| 6 | TA109 | AS31 | 17 | 0.84 | 6 | 0.8 | 14 | 0.85 | 12 | | 0.86 | 7 | | 0.82 | 13 | | 0.82 |  |
|  |  | TA109 | 17 | 0.81 | 5 | 0.67 | 14 | 0.8 | 12 | | 0.82 | 6 | | 0.81 | 12 | | 0.84 |  |
| 6 | TA1 | AS8 | 6 | 0.5 | 2 | 0.48 | 5 | 0.45 | 3 | | 0.39 | 3 | | 0.52 | 4 | | 0.57 |  |
|  |  | AS7 | 6 | 0.34 | 2 | 0.18 | 4 | 0.33 | 2 | | 0.32 | 3 | | 0.21 | 4 | | 0.43 |  |
|  |  | TA1 | 18 | 0.89 | 7 | 0.89 | 14 | 0.88 | 10 | | 0.87 | 11 | | 0.89 | 14 | | 0.89 |  |
| 10 | TA40 | TA40 | 21 | 0.89 | 8 | 0.95 | 20 | 0.88 | 11 | | 0.87 | 12 | | 0.93 | 18 | | 0.9 |  |
|  |  | AS25 | 17 | 0.83 | 6 | 0.85 | 14 | 0.85 | 12 | | 0.82 | 11 | | 0.84 | 11 | | 0.77 |  |
|  |  | B7M19 | 6 | 0.56 | 4 | 0.64 | 5 | 0.61 | 3 | | 0.49 | 3 | | 0.36 | 6 | | 0.52 |  |
| 11 | ARA2 | ARA2 | 12 | 0.83 | 5 | 0.76 | 12 | 0.82 | 9 | | 0.87 | 6 | | 0.75 | 9 | | 0.86 |  |
|  |  | AS1 | 4 | 0.28 | 2 | 0.3 | 3 | 0.25 | 3 | | 0.37 | 4 | | 0.46 | 3 | | 0.24 |  |
|  |  | AS2 | 10 | 0.77 | 3 | 0.62 | 9 | 0.78 | 7 | | 0.79 | 6 | | 0.73 | 8 | | 0.74 |  |
|  |  | AS3 | 11 | 0.87 | 7 | 0.92 | 11 | 0.87 | 10 | | 0.87 | 7 | | 0.82 | 10 | | 0.85 |  |
| 12 | PfPK2 | PFPK2 | 19 | 0.88 | 6 | 0.85 | 14 | 0.88 | 12 | | 0.88 | 8 | | 0.85 | 17 | | 0.9 |  |
|  |  | AS32 | 14 | 0.7 | 3 | 0.62 | 9 | 0.71 | 8 | | 0.71 | 7 | | 0.74 | 9 | | 0.68 |  |
| 12 | PfG377 | AS34 | 5 | 0.54 | 2 | 0.53 | 4 | 0.54 | 4 | | 0.55 | 4 | | 0.61 | 4 | | 0.5 |  |
|  |  | PFG377 | 7 | 0.64 | 2 | 0.55 | 7 | 0.64 | 4 | | 0.61 | 5 | | 0.63 | 7 | | 0.66 |  |
| 13 | TA60 | AS15 | 11 | 0.85 | 5 | 0.86 | 10 | 0.85 | 11 | | 0.87 | 8 | | 0.85 | 11 | | 0.86 |  |
|  |  | TA60 | 12 | 0.85 | 7 | 0.91 | 12 | 0.85 | 7 | | 0.83 | 8 | | 0.87 | 10 | | 0.84 |  |
|  |  | mean | 11.6 | 0.71 | 4.5 | 0.68 | 10.2 | 0.7 | 7.6 | | 0.69 | 6.3 | | 0.69 | 9.2 | | 0.71 |  |

^*^Chr is Chromosomal location of the microsatellites; ^†^*A* is number of unique alleles and ^‡^*He* the mean expected heterozygosity
