# Supplementary figures and images for: Confirmation of the absence of local transmission and geographic assignment of imported falciparum malaria cases to China using microsatellite panel
Source: Malar J. 2020 Jul 13;19:244. doi: 10.1186/s12936-020-03316-3 (PMC7359230; doi:10.1186/s12936-020-03316-3)

## Slide 1
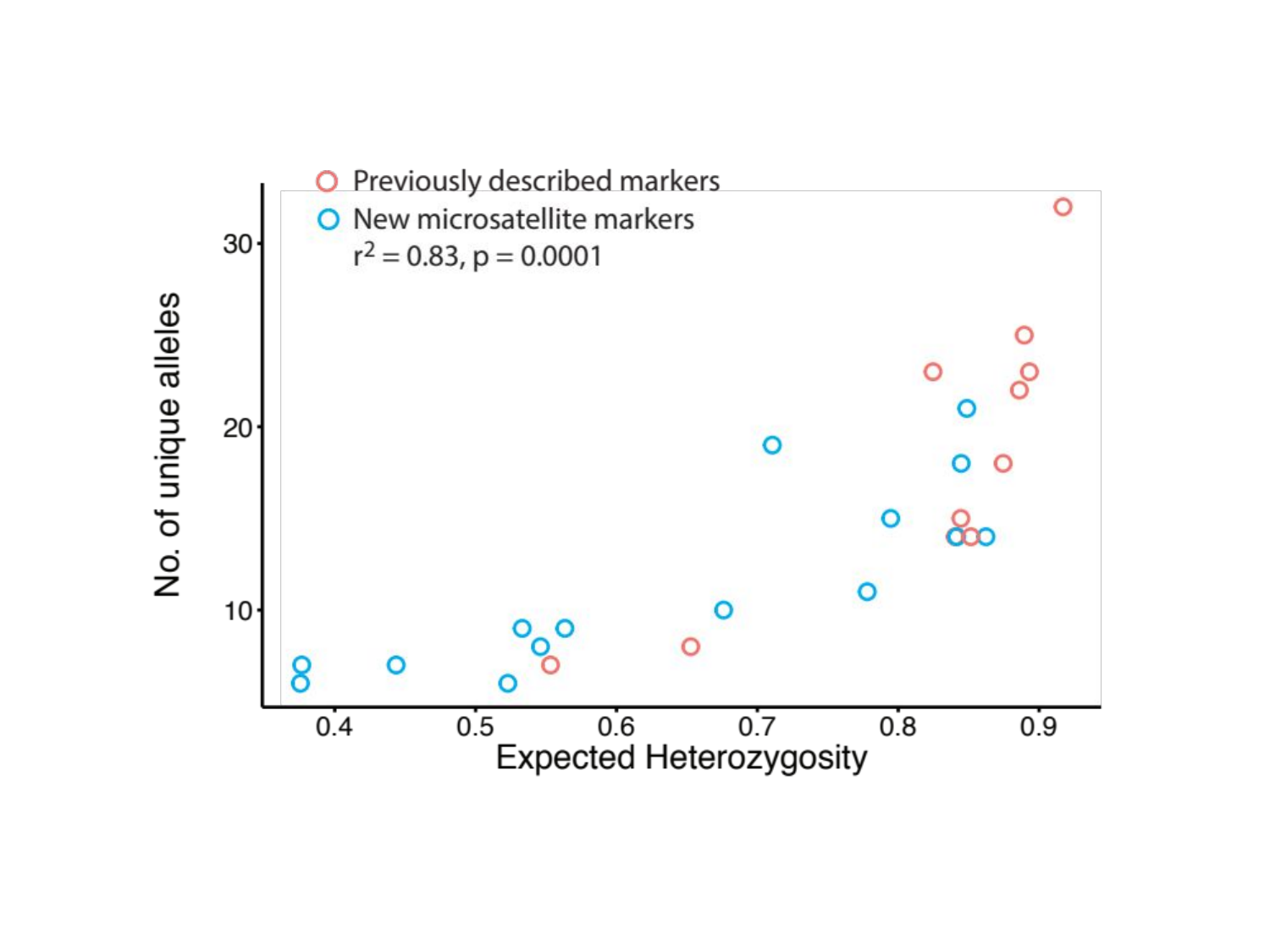

Supplement: Supplementary file 4 — Additional file 4. Relationship between the number of unique alleles and expected heterozygosity in 602 Plasmodium falciparum cases imported to Jiangsu Province from 26 African countries. Previously described markers are shown in red and new markers are shown in blue. [file 12936_2020_3316_MOESM4_ESM.pptx]

## Slide 1
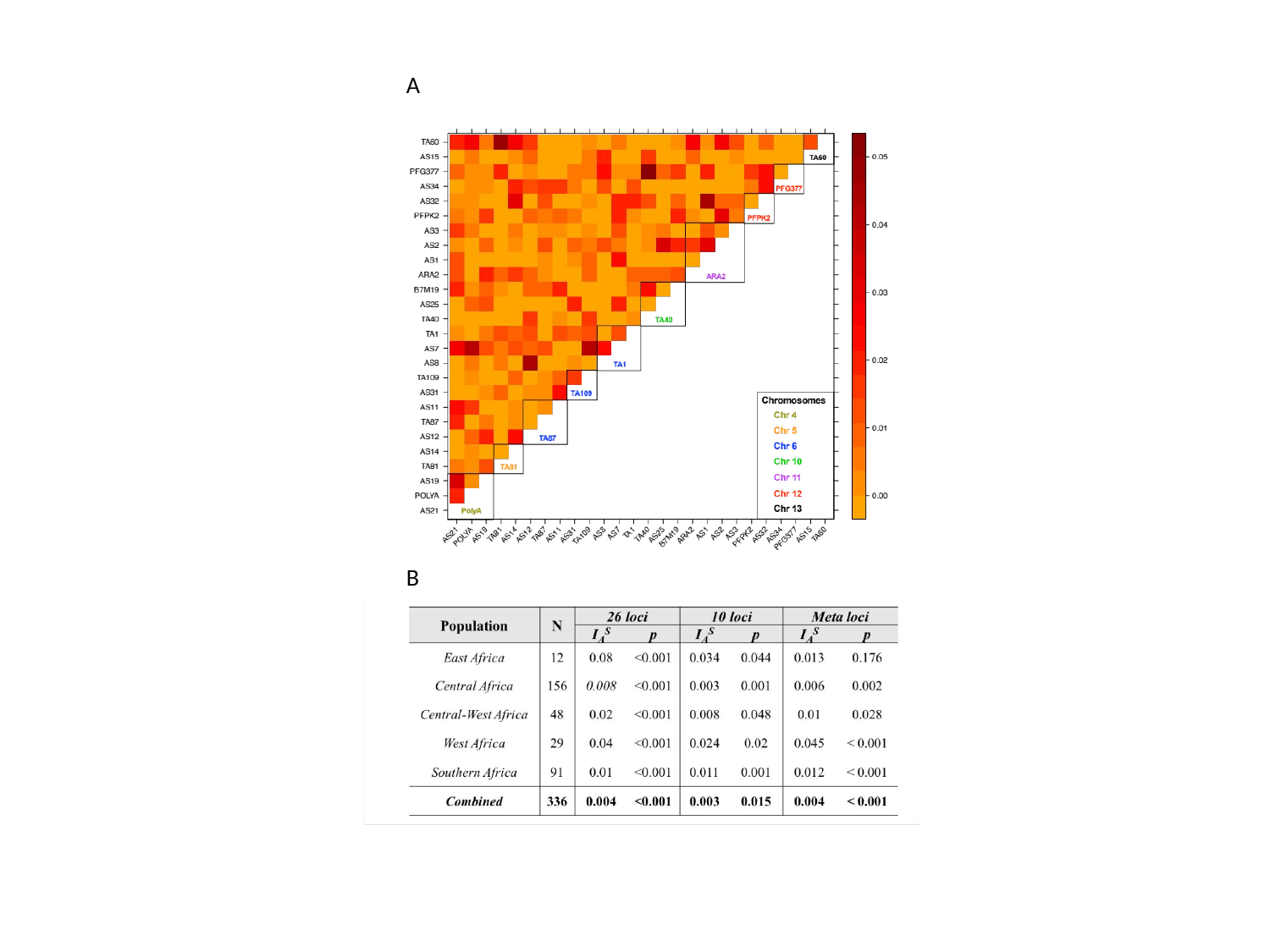

A
B

Supplement: Supplementary file 5 — Additional file 5. Pair-wise linkage disequilibrium between 26 microsatellite markers. Pair-wise index of associations (IA) is indicated. B. Estimates of multi-locus linkage disequilibrium in 336 Plasmodium falciparum isolates imported from five different regions of sub-Saharan Africa to Jiangsu Province, China IAS is the standardized index of association. [file 12936_2020_3316_MOESM5_ESM.pptx]

## Slide 1
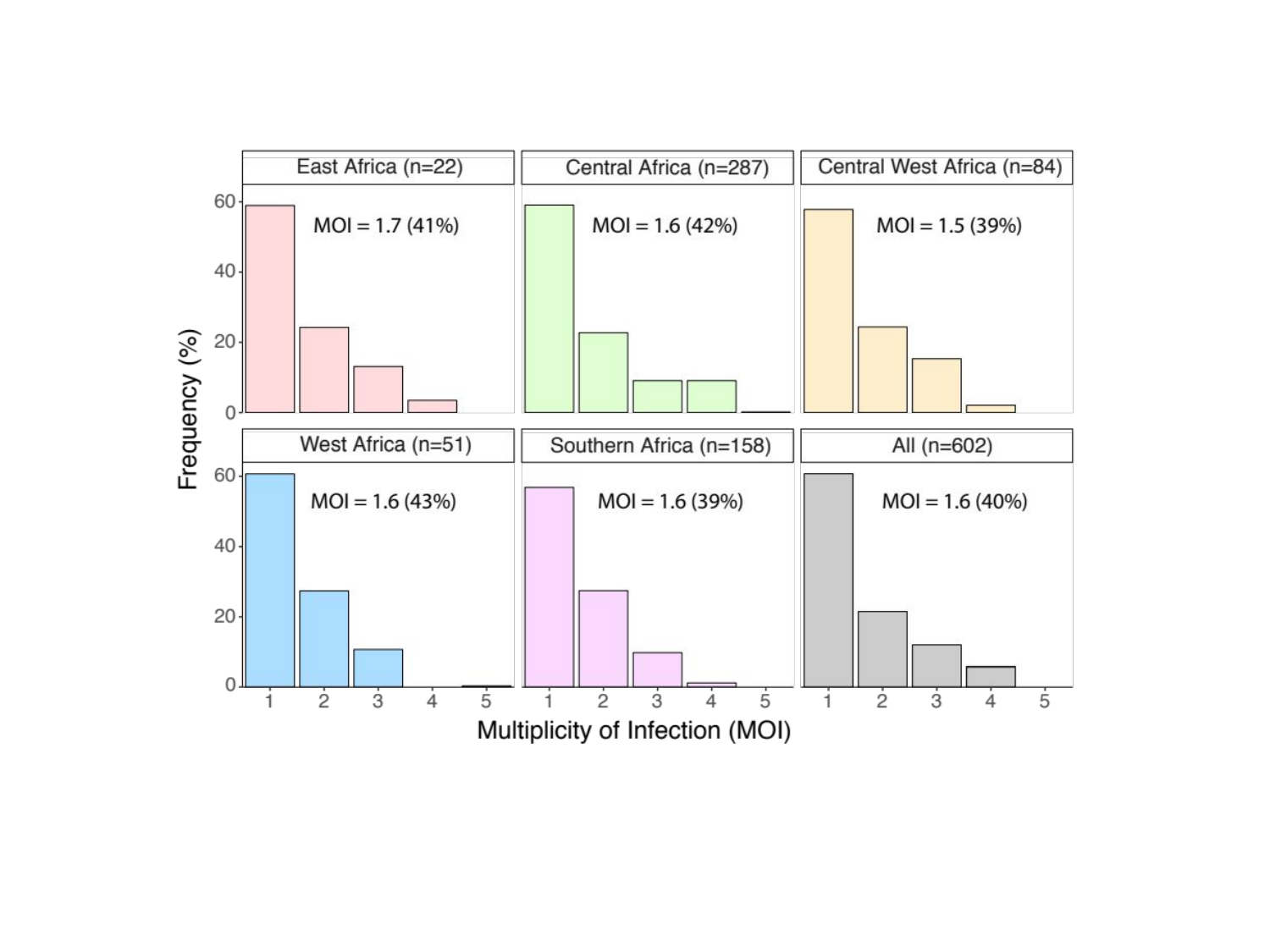

Supplement: Supplementary file 6 — Additional file 6. Mean multiplicity of infection (MOI) and percentage of polyclonal samples by the geographic origin of imported infections to the Jiangsu Province, China. [file 12936_2020_3316_MOESM6_ESM.pptx]

## Slide 1
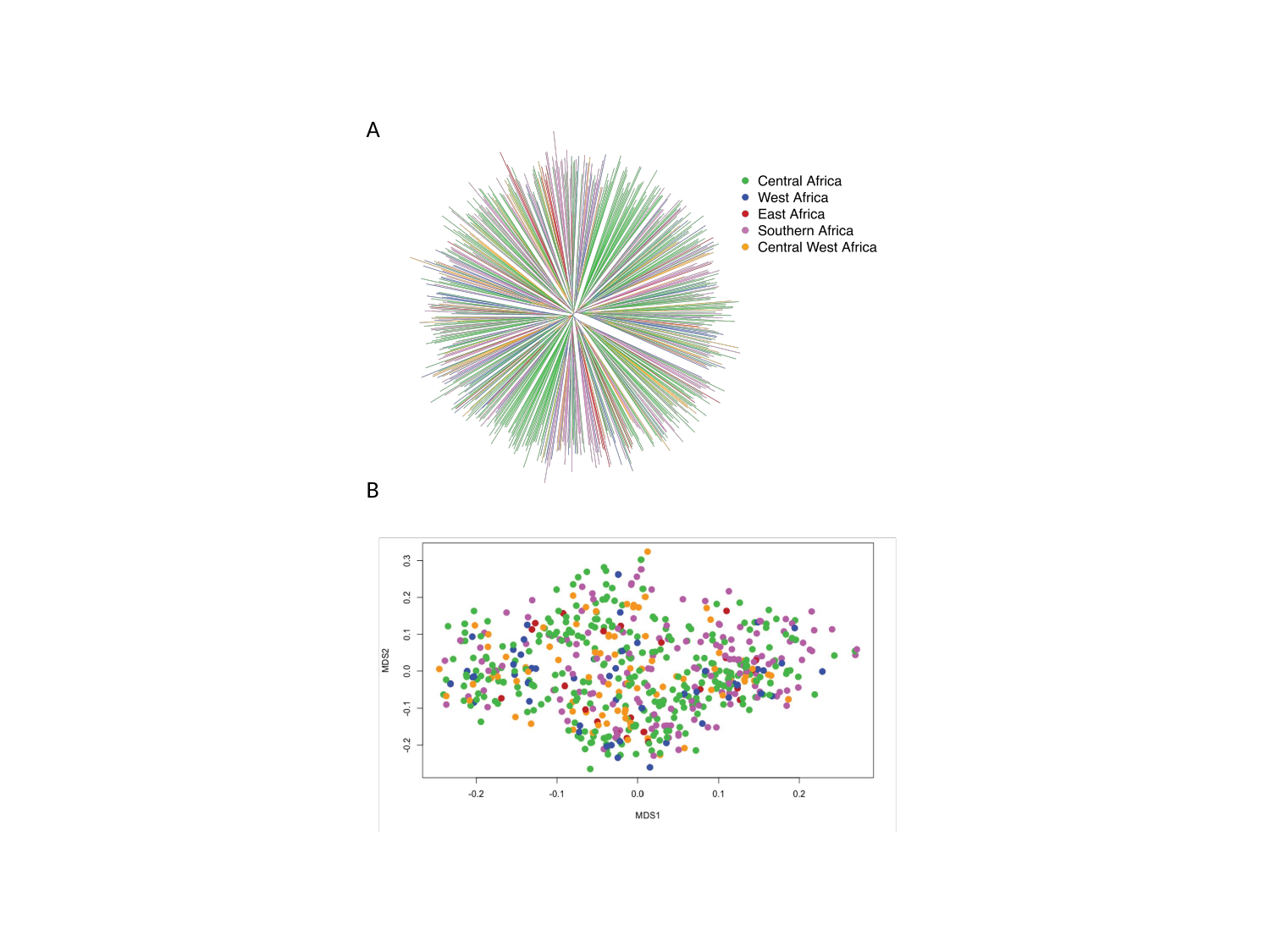

A
B

Supplement: Supplementary file 7 — Additional file 7. A. Neighbour-joining tree showing genetic relatedness of 602 imported Plasmodium falciparum isolates, branches are coloured according to region of origin of the imported case. B. Plot of second principal component against the first, computed from a multidimensional scaling based on the same distance matrix used for the tree shown in panel A. [file 12936_2020_3316_MOESM7_ESM.pptx]
